# SARS-CoV-2 Infection as a Cause of Acute Pancreatitis in a Child—A Case Report

**DOI:** 10.3390/pediatric13040065

**Published:** 2021-10-01

**Authors:** Natalia Kopiczko, Kamila Kwiatek-Średzińska, Mirosława Uścinowicz, Monika Kowalczuk-Krystoń, Dariusz Marek Lebensztejn

**Affiliations:** Department of Pediatrics, Gastroenterology, Hepatology, Nutrition and Allergology, Medical University of Bialystok, 15-274 Białystok, Poland; kamila.kwiatek90@gmail.com (K.K.-Ś.); mirusc@o2.pl (M.U.); monika.kowalczuk@udsk.pl (M.K.-K.); lebensztejn@hoga.pl (D.M.L.)

**Keywords:** children, acute pancreatitis, COVID-19, SARS-CoV-2

## Abstract

The novel coronavirus disease (COVID-19) was detected for the first time in China in December 2019. Soon after it was declared a pandemic. Main symptoms include fever, dyspnea, cough, muscle pain, headache, anosmia and ageusia, however a growing body of evidence shows that other organs can be affected. Gastrointestinal manifestations have been observed in a considerable number of patients and include abdominal pain, diarrhea and vomiting. The involvement of liver as well as pancreas has been also described, however there are only a few cases of acute pancreatitis reported in patients with COVID-19. Therefore, we present a case of 6-year-old child with mild acute pancreatitis and COVID-19 pneumonia.

## 1. Introduction

**Figure 1:**
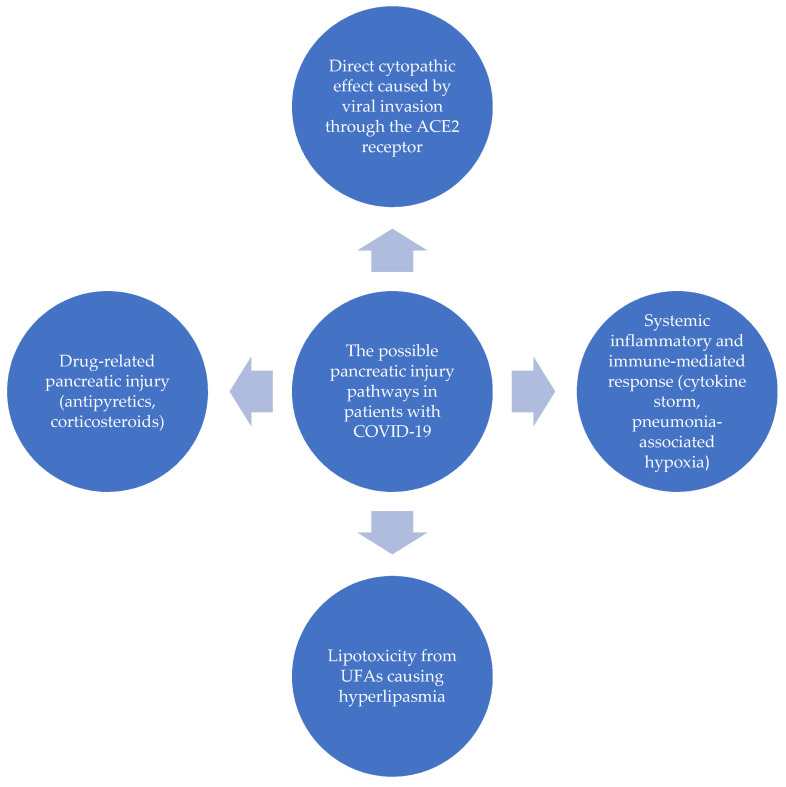
The physiopathology of pancreatic involvement in the course of COVID-19 disease.

The coronavirus disease 2019 (COVID-19) is an infectious disease caused by Severe Acute Respiratory Syndrome Coronavirus 2 (SARS-CoV-2) and was described for the first time in December 2019 in Wuhan, China [1]. A rapid spread of this disease was observed and it was declared a pandemic on 11 March 2020 [2]. According to WHO, there are 99,363,697 confirmed cases of COVID-19, including 2,135,959 deaths as on 27 January 2021 [3]. The most common symptoms of COVID-19 include fever, dyspnea, cough, muscle pain, headache, sore throat and loss of taste or smell. However, more organs have been observed to be affected by SARS-CoV-2 over time. Involvement of nervous, digestive, renal and cardiovascular systems were described in the course of COVID-19 [4,5]. Gastrointestinal (GI) manifestations were found in 10–15% of adult COVID-19 patients and include abdominal pain, diarrhea and vomiting [6]. Moreover, liver and pancreas involvement has been also linked to SARS-CoV-2 infection. Among others, acute pancreatitis (AP) is thought to be associated with COVID-19 [5,7,8,9]. Diagnostic criteria for AP include: abdominal pain, elevated serum lipase/amylase activity (at least three times the normal level) and characteristic findings in imaging studies (CT/MRI/ultrasonography). AP may be diagnosed when two of three criteria are met. Biliary abnormalities are the most common etiology for AP, both in adults and children, however other possible causes should be included in differential diagnosis (genetic, autoimmune, metabolic disorders, infectious agents, pancreatic abnormalities, drug-induced injury) [10,11]. It is well known that AP may also occur secondarily to viral infection-cytomegalovirus, Coxsackie virus, Epstein–Barr virus, hepatitis A, B, E, herpes simplex, HIV, mumps, measles and varicella-zoster viruses were described as possible causative agents. Therefore, it is suspected that SARS-CoV-2 may also cause pancreas injury. The coronavirus, thanks to very similar spike protein 3-D structure, uses the angiotensin-converting enzyme 2 (ACE 2) receptor to gain cellular entry to host cells. ACE 2 receptors are widely expressed in epithelial tissue of the lung, as well as gastrointestinal system including the pancreas [12,13]. Potential mechanisms involved in the physiopathology of pancreatic injury in patients with COVID-19 include the direct cytopathic effects caused by entering the cell through ACE 2 receptors, indirect systemic inflammatory and immune-mediated cellular responses, virus-related lipotoxicity from unsaturated fatty acids (UFAs) causing hiperlipasemia and drug-induced pancreatic damage caused by non-steroidal anti-inflammatory drugs (NSAIDs) and corticosteroids Figure 1 [7,13]. The histopathological pancreatic changes associated with SARS-CoV-2 infection are as follows: degeneration of the islet cells, pancreatitis (from microscopic acute inflammation to necrotic-hemorrhagic lesions) [7,14]. In the literature there are cases of acute pancreatitis in the course of COVID-19, but only a few cases have been described in children [15,16,17,18]. The coexistence of AP and SARS-CoV-2 infection causes increased systemic inflammatory response, contributed to the worsening of the patient’s clinical state. COVID-19 positive patients with AP had a greater risk of developing severe AP, local complications and persistent organ failure. Their hospital stay was longer, the intensive care unit admission rate higher and mortality increased compared to COVID-19 negative patients with AP [19,20]. We report a case of a child with mild acute pancreatitis and COVID-19 pneumonia.

COVID-19—coronavirus disease 2019, ACE 2—angiotensin-converting enzyme 2, UFs—unsaturated fatty acids.

**Case Report:**

A 6-year-old, previously healthy female was admitted to the Department due to the first episode of acute pancreatitis. The patient was transferred from another medical center, where she had presented with epigastric pain, vomiting as well as three-day history of mild cold-like symptoms. Laboratory tests that had been done there revealed leukocytosis with neutrophilia, significantly elevated amylase and lipase activity (910 IU/L—normal range: 28–100 IU/L, 4159 IU/L—normal range: 13–60 IU/L, respectively), but her abdominal ultrasonography was normal. On admission the patient appeared uncomfortable, complained of abdominal pain, but her vital signs were stable and within normal values. Examination revealed abdominal distension and tenderness to palpation of the epigastric region, signs of dehydration, tachycardia and eye redness after exotropia surgery (October 2020). Laboratory studies demonstrated markedly elevated total and pancreas-specific serum amylase and lipase activity, leukocytosis with neutrophilia, increased concentration of D-dimers, interleukin-6 and fibrinogen. Other blood count parameters, C-reactive protein (CRP), procalcitonin (PCT), liver and kidney function tests were within normal range. Transabdominal ultrasonography demonstrated moderate pancreas enlargement (head—21 mm, body—12 mm, tail—20 mm), reduced echogenicity of the organ and moderate amount of free fluid in the lesser pelvis and the right iliac fossa, cholelithiasis was ruled out. In differential diagnosis trauma, biliary and pancreatic abnormalities, infections (hepatitis B, C, cytomegalovirus, Epstein-Barr virus, rotavirus, adenovirus, norovirus, Chlamydia pneumoniae, Mycoplasma pneumoniae, Toxocara, Toxoplasma, parasites in stool), metabolic disorders (hypercalcemia, hypertriglyceridemia, diabetic ketoacidosis), presence of underlying systemic disease, autoimmune and medication-associated pancreatitis were ruled out. Genetic testing was not performed because it was the first episode of AP. Due to the presence of cold-like symptoms the girl was tested for COVID-19 and her nasopharyngeal swab (using reverse transcriptase–polymerase chain reaction (RT-PCR) method) was positive for SARS-CoV-2. Chest radiography showed accentuated picture of the stroma in the perihilar region of the left lung and lung parenchyma consolidations. Taking all this into consideration, SARS-CoV-2 infection was confirmed as potential cause of AP. Patient’s parent consent was taken for this case report.

The patient was consulted by a cardiologist due to observed tachycardia (130–150 heart beats/minute)-cardiac enzymes were within normal range and electrocardiogram did not reveal any abnormalities. Parameters of hemostasis as well as inflammatory markers were analyzed by a hematologist together with infectious diseases specialists who recommended introducing low-molecular-weight heparin (enoxaparin 1 mg/kg once daily) due to significantly elevated level of D-dimers. In the first two days of hospitalization enteral nutrition was withheld, the patient was given intravenous fluids, non-opioid analgesics and enoxaparin in the recommended dose. Realimentation was started on the third day of hospitalization. Initially the patient was fed with protein liquid peptide-based diet (Peptamen), then low-fat diet was administered with good tolerance. Abdomen–pelvis contrast enhanced computed tomography (CECT) and chest high-resolution computed tomography (HRCT) were performed on the7th day of hospitalization. Abdomen-pelvis CECT demonstrated moderate pancreas enlargement (head—25 mm, body—13 mm, tail—19 mm), heterogenous contrast enhancement of the organ and hyperdense area near the pancreas head size 22 × 19 mm visible in arterial phase—most likely the ascending duodenum with thickened wall up to 7.5 mm, no peripancreatic fluid collection was showed. Chest HRCT demonstrated accentuated picture of the lung stroma and subpleural pulmonary nodule in segment 10 of the left lung. On discharge a downward trend in pancreatic enzymes activity was achieved. Dynamics of selected laboratory parameters (pancreatic enzymes, inflammatory markers, potential predictors of the severity of AP) is presented in Table 1. The patient was educated about low-fat diet and need for Peptamen supplementation by a dietitian and after eight days was discharged home. 

## 2. Discussion

Gastrointestinal involvement has been reported in patients with COVID-19 with a prevalence of approximately 10% in both adults and children [21,22]. Most common symptoms include diarrhea, nausea or vomiting and abdominal pain, however recent evidence shows that other organs such as liver or pancreas can also be affected [5,7]. The angiotensin-converting enzyme 2 receptor has been suggested to play a central role in patomechanism of GI tract symptoms as it was established a functional receptor for SARS-CoV-2 enabling it to enter the host cells. ACE 2 receptor is mostly expressed in alveolar cells of the lungs, however it was also found in gastrointestinal tract [23] Interestingly, increased expression of ACE 2 receptor was found in endocrine cells of the pancreas and suggested to be a cause of acute diabetes mellitus in patients with SARS-CoV infection during its outbreak in 2002–2004 [24]. Presence of ACE 2 receptor in pancreatic cells may also serve as a potential entry for SARS-CoV-2 and lead to acute pancreatitis in the course of COVID-19. We presented a case of a 6-year-old girl with a diagnose of acute pancreatitis in the course of COVID-19. Her main complaints were vomiting and epigastric pain, she had a three-day history of cold-like symptoms, however there were no signs of respiratory distress or fever. She was unvaccinated against COVID-19 and her previous SARS-CoV-2 infection status was negative. The severity of AP was classified as mild because no organ failure and local or systemic complications were observed. So far there are only a few case reports regarding the coexistence of SARS-CoV-2 infection and pancreatitis in pediatric patients. Samies et al. reported three cases of acute pancreatitis in children younger than 16 years old who tested positive for SARS-CoV-2. Similar to our patient, the course of AP was rather mild and there were no signs of respiratory distress. Only one girl had a single gallstone, that could potentially suggest biliary etiology of the disease, in two patients no other possible causes could be found [18]. Suchman et al. presented a retrospective analysis of 8159 pediatric patients of which 112 tested positive for SARS-CoV-2. Thirteen patients were diagnosed with acute pancreatitis (prevalence 0.16%) and only two of them had COVID-19. According to their study, the prevalence of AP in children with COVID-19 was higher than in the non-COVID-19 population (1.8% vs. 0.14%, respectively) [15]. Other reports include a case of a 7-year-old girl that developed necrotizing pancreatitis two weeks prior to her positive test for SARS-CoV-2 infection and a case of a 10-year-old patient with an acute pancreatitis as a presentation of multisystem inflammatory syndrome in children (MIS-C) [16,17]. In most children AP has a non-fulminant course that resolves without complications with supportive care [11]. Additionally, when COVID-associated, the manifestation is indolent due to an immature immune system. To sum up, since pancreas involvement is observed in patients with COVID-19, it seems important to assess amylase and lipase, especially when abdominal pain or vomiting are among reported symptoms. 

## Figures and Tables

**Table 1:** Dynamics of selected laboratory parameters (pancreatic enzymes, inflammatory markers, potential predictors of the severity of AP).

Parameter (Normal Range)	On Admission	After 24 h	After 48 h	After 72 h	On Discharge
Amylase (28–100 IU/L)	1083	1224	745	272	141
Pancreatic amylase (13–53 U/L)	1124		685	236	111
Lipase (13–60 IU/L)	4250	3225	1439	516	281
CRP (0–5 mg/L)	<0.6	3.31	8.47	5.05	<0.6
PCT (0–0.5 ng/mL)	<0.05		<0.05		
WBC (4–12 × 10^3^/µL)	14.4	14.56	14.36	7.17	5.03
HGB (12–15.5 g/dL)	12.4	11.8	10.4	10.5	11.1
PLT (140–450 × 10^3^/µL)	480	470	477	407	471
D-dimers (0–500 ng/mL)	1639	3934		8403	757
Fibrinogen (200–360 mg/dL)			409	306	120
IL-6 (0–7 pg/mL)			11.18		4.42
Albumin (3.8–5.4 g/dL)	4.32	4.39	3.89		
Ca (2.19–2.69 mmol/L)	2.33	2.38	2.43		
Creatinine (0.32–0.59 mg/dL)	0.36	0.43	0.39	0.32	0.3
Urea (0–50 mg/dL)	27	23	23		

CRP—C-reactive protein, PCT—procalcitonin, WBC—white blood cells, HGB—haemoglobin, PLT—platelets, IL-6—interleukin 6, Ca—calcium.

## Data Availability

Data available via email.

## References

[1] WHO Pneumonia of Unknown Cause—China. 5 January 2020. https://www.who.int/csr/don/05-january-2020-pneumonia-of-unkown-cause-china/en/2020.

[2] WHO WHO Director-General’s Opening Remarks at the Media Briefing on COVID-19. 11 March 2020. https://www.who.int/director-general/speeches/detail/who-director-general-s-opening-remarks-at-the-media-briefing-on-covid-19---11-march-20202020.

[3] WHO WHO Coronavirus Disease (COVID-19) Dashboard. 27 January 2021. https://covid19.who.int.

[4] Machhi J., Herskovitz J., Senan A.M., Dutta D., Nath B., Oleynikov M.D., Blomberg W.R., Meigs D.D., Hasan M., Patel M. (2020). The Natural History, Pathobiology, and Clinical Manifestations of SARS-CoV-2 Infections. J. Neuroimmune Pharmacol..

[5] Mao R., Qiu Y., He J.S., Tan J.Y., Li X.H., Liang J., Shen J., Zhu L.R., Chen Y., Iacucci M. (2020). Manifestations and prognosis of gastrointestinal and liver involvement in patients with COVID-19: A systematic review and meta-analysis. Lancet Gastroenterol. Hepatol..

[6] Pan L., Mu M., Yang P., Sun Y., Wang R., Yan J., Li P., Hu B., Wang J., Hu C. (2020). Clinical Characteristics of COVID-19 Patients With Digestive Symptoms in Hubei, China: A Descriptive, Cross-Sectional, Multicenter Study. Am. J. Gastroenterol..

[7] Samanta J., Gupta R., Singh M.P., Patnaik I., Kumar A., Kochhar R. (2020). Coronavirus disease 2019 and the pancreas. Pancreatology.

[8] Barlass U., Wiliams B., Dhana K., Adnan D., Khan S.R., Mahdavinia M., Bishehsari F. (2020). Marked Elevation of Lipase in COVID-19 Disease: A Cohort Study. Clin. Transl. Gastroenterol..

[9] Bansal P., Margekar S.L., Suman V., Sud R., Meena S., Sharma A.K., Islam S.Y., Gurtoo A., Agrawal A., Pangtey G.S. (2020). Pancreatic Injury in COVID-19 Patients. J. Assoc. Physicians India.

[10] Banks P.A., Bollen T.L., Dervenis C., Gooszen H.G., Johnson C.D., Sarr M.G., Tsiotos G.G., Vege S.S. (2013). Classification of acute pancreatitis—2012: Revision of the Atlanta classification and definitions by international consensus. Gut.

[11] Párniczky A., Abu-El-Haija M., Husain S., Lowe M., Oracz G., Sahin-Tóth M., Szabó F.K., Uc A., Wilschanski M., Witt H. (2018). EPC/HPSG evidence-based guidelines for the management of pediatric pancreatitis. Pancreatology.

[12] Zou X., Chen K., Zou J., Han P., Hao J., Han Z. (2020). Single-cell RNA-seq data analysis on the receptor ACE2 expression reveals the potential risk of different human organs vulnerable to 2019-nCoV infection. Front. Med..

[13] Patel K.P., Patel P.A., Vunnam R.R., Hewlett A.T., Jain R., Jing R., Vunnam S.R. (2020). Gastrointestinal, hepatobiliary, and pancreatic manifestations of COVID-19. J. Clin. Virol..

[14] Caramaschi S., Kapp M.E., Miller S.E., Eisenberg R., Johnson J., Epperly G., Maiorana A., Silvestri G., Giannico G.A. (2021). Histopathological findings and clinicopathologic correlation in COVID-19: A systematic review. Mod. Pathol..

[15] Suchman K., Raphael K.L., Liu Y., Wee D., Trindade A.J., Northwell COVID-19 Research Consortium (2021). Acute pancreatitis in children hospitalized with COVID-19. Pancreatology.

[16] Stevens J.P., Brownell J.N., Freeman A.J., Bashaw H. (2020). COVID-19-associated Multisystem Inflammatory Syndrome in Children Presenting as Acute Pancreatitis. J. Pediatr. Gastroenterol. Nutr..

[17] Alloway B.C., Yaeger S.K., Mazzaccaro R.J., Villalobos T., Hardy S.G. (2020). Suspected case of COVID-19-associated pancreatitis in a child. Radiol. Case Rep..

[18] Samies N.L., Yarbrough A., Boppana S. (2020). Pancreatitis in Pediatric Patients with COVID-19. J. Pediatr. Infect. Dis. Soc..

[19] Karaali R., Topal F. (2021). Evaluating the effect of SARS-Cov-2 infection on prognosis and mortality in patients with acute pancreatitis. Am. J. Emerg. Med..

[20] Pandanaboyana S., Moir J., Leeds J.S., Oppong K., Kanwar A., Marzouk A., Belgaumkar A., Gupta A., Siriwardena A.K., Haque A.R. (2021). SARS-CoV-2 infection in acute pancreatitis increases disease severity and 30-day mortality: COVID PAN collaborative study. Gut.

[21] Jin X., Lian J.S., Hu J.H., Gao J., Zheng L., Zhang Y.M., Hao S.R., Jia H.Y., Cai H., Zhang X.L. (2020). Epidemiological, clinical and virological characteristics of 74 cases of coronavirus-infected disease 2019 (COVID-19) with gastrointestinal symptoms. Gut.

[22] Rokkas T. (2020). Gastrointestinal involvement in COVID-19: A systematic review and meta-analysis. Ann. Gastroenterol..

[23] Bourgonje A.R., Abdulle A.E., Timens W., Hillebrands J.L., Navis G.J., Gordijn S.J., Bolling M.C., Dijkstra G., Voors A.A., Osterhaus A.D. (2020). Angiotensin-converting enzyme 2 (ACE2), SARS-CoV-2 and the pathophysiology of coronavirus disease 2019 (COVID-19). J. Pathol..

[24] Yang J.K., Lin S.S., Ji X.J., Guo L.M. (2010). Binding of SARS coronavirus to its receptor damages islets and causes acute diabetes. Acta Diabetol..

